# Supplementary material for: Morphological Evaluation of Transscleral Laser Retinopexy in Rabbits: Comparison of Optical Coherence Tomography and Histologic Examinations
Source: Vet Sci. 2023 Aug 23;10(9):535. doi: 10.3390/vetsci10090535 (PMC10534503; doi:10.3390/vetsci10090535)
Supplement: Supplementary file 1 [file vetsci-10-00535-s001.zip › vetsci-2488929-supplementary.pdf]

| Variable | Time | Estimate | ± SE | Df | Time ratio | P-values |
|---|---|---|---|---|---|---|
| Left side (LS) | J0 - J1 | 23.75 | 15.3 | 37.8 | -1.55 | 0.176 |
| | **J0 - J15** | **86.63** | **17.7** | **37.9** | **4.89** | **<0.001** |
| | **J0 - J21** | **107.14** | **17.7** | **38.2** | **6.04** | **<0.001** |
| | **J0 - J42** | **106.14** | **20.3** | **38.1** | **5.23** | **<0.001** |
| | **J0 - J7** | **71.05** | **16.3** | **37.9** | **4.37** | **<0.001** |
| | **J1 - J15** | **110.38** | **17.7** | **38.0** | **6.24** | **<0.001** |
| | **J1 - J21** | **130.89** | **17.7** | **38.2** | **7.38** | **<0.001** |
| | **J1 - J42** | **129.89** | **20.3** | **38.2** | **6.40** | **<0.001** |
| | **J1 - J7** | **94.80** | **16.3** | **37.9** | **5.83** | **<0.001** |
| | J15 - J21 | 20.50 | 19.8 | 38.3 | 1.03 | 0.385 |
| | J15 – J42 | 19.51 | 22.2 | 38.2 | 0.88 | 0.434 |
| | J15 – J7 | -15.59 | 18.5 | 38.0 | -0.84 | 0.434 |
| | J21 – J42 | -0.99 | 22.2 | 38.4 | -0.05 | 0.965 |
| | J21 – J7 | -36.09 | 18.6 | 38.3 | -1.95 | 0.099 |
| | J42 – J7 | 35.10 | 21.0 | 38.2 | -1.67 | 0.155 |
| | | | | | | |
| Center (CS) | J0 - J1 | -59.00 | 27.9 | 37.9 | -2.12 | 0.061 |
| | **J0 - J15** | **168.44** | **32.2** | **38.0** | **5.23** | **< 0.001** |
| | **J0 - J21** | **193.79** | **32.2** | **38.1** | **6.01** | **< 0.001** |
| | **J0 - J42** | **185.35** | **36.9** | **38.1** | **5.02** | **< 0.001** |
| | **J0 - J7** | **109.39** | **29.6** | **38.0** | **3.70** | **0.001** |
| | **J1 - J15** | **227.44** | **32.2** | **38.0** | **7.06** | **< 0.001** |
| | **J1 - J21** | **252.79** | **32.2** | **38.1** | **7.84** | **< 0.001** |
| | **J1 - J42** | **244.35** | **36.9** | **38.1** | **6.62** | **< 0.001** |
| | **J1 - J7** | **168.39** | **29.6** | **38.0** | **5.69** | **< 0.001** |
| | J15 - J21 | 25.35 | 36.0 | 38.2 | 0.70 | 0.561 |
| | J15 - J42 | 16.91 | 40.3 | 38.1 | 0.42 | 0.725 |
| | J15 - J7 | -59.05 | 33.7 | 38.0 | -1.75 | 0.110 |
| | J21 - J42 | -8.44 | 40.3 | 38.2 | -0.21 | 0.835 |
| | J21 - J7 | -84.40 | 33.7 | 38.2 | -2.50 | 0.028 |
| | J42 - J7 | -75.96 | 38.2 | 38.1 | -1.99 | 0.074 |
| | | | | | | |
| Right side (RS) | J0 - J1 | -23.75 | 13.2 | 37.7 | -1.80 | 0.110 |
| | **J0 - J15** | **77.26** | **15.3** | **37.9** | **5.06** | **< 0.001** |
| | **J0 - J21** | **100.64** | **15.3** | **38.3** | **6.57** | **< 0.001** |
| | **J0 - J42** | **101.76** | **17.5** | **38.2** | **5.80** | **< 0.001** |
| | **J0 - J7** | **65.79** | **14.0** | **37.9** | **4.69** | **< 0.001** |
| | **J1 - J15** | **101.00** | **15.3** | **38.0** | **6.61** | **< 0.001** |
| | **J1 - J21** | **124.39** | **15.3** | **38.3** | **8.12** | **< 0.001** |
| | **J1 - J42** | **125.51** | **17.5** | **38.2** | **7.15** | **< 0.001** |
| | **J1 - J7** | **89.54** | **14.0** | **37.9** | **6.38** | **< 0.001** |

| | | | | | | |
|---|---|---|---|---|---|---|
| | J15 - J21 | 23.39 | 17.1 | 38.4 | 1.37 | 0.225 |
| | J15 - J42 | 24.50 | 19.2 | 38.3 | 1.28 | 0.241 |
| | J15 - J7 | -11.47 | 16.0 | 38.1 | -0.72 | 0.512 |
| | J21 - J42 | 1.11 | 19.2 | 38.5 | 0.06 | 0.954 |
| | J21 - J7 - | -34.86 | 16.0 | 38.4 | -2.17 | 0.060 |
| | J42 - J7 | -35.97 | 18.2 | 38.3 | -1.98 | 0.082 |

**Table S1.** Results of post-hoc tests, comparing the different modalities of the time variable. P values were corrected by the Benjamini-Hochberg method. In bold: significant differences.

To read the table, if the P-values are significant (in bold), it means that there is a significant difference between the two times compared. The significance of the associated estimate should be considered. If the sign is negative, then the first time of the comparison is less than the second time. If the sign is positive, the first Time is greater than the second Time.
